# The hidden cross talk between bone and tissues through bone turnover

**DOI:** 10.1515/almed-2023-0160

**Published:** 2023-12-12

**Authors:** María Luisa González-Casaus

**Affiliations:** Laboratory Medicine, University Hospital La Paz Madrid, Madrid, Spain

**Keywords:** endocrine activity, sclerostin, osteocalcin, bone turnover, Wnt/β-catenin

## Abstract

Bone is more than a reservoir of calcium and phosphorus. Its lacuno-canalicular arrangement provides an important pathway for exchange with circulation and currently, the skeleton is considered a large endocrine organ with actions that go beyond the control of calcium-phosphorus balance mediated by fibroblastic growth factor 23 (FGF23). Parallel to the modulating effect of adipokines on bone turnover, certain bone proteins, such as osteocalcin and sclerostin, play a counter-regulatory role on energy metabolism, probably in an attempt to ensure its high energy requirement for bone turnover. In this crosstalk between bone and other tissues, especially with adipose tissue, canonical Wnt/β-catenin signaling is involved and therefore, sclerostin, an osteocyte derived protein that inhibits this signalling, emerges as a potential biomarker. Furthermore, its involvement in diverse pathologic conditions supports sclerostin as a therapeutic target, with an anti-sclerostin antibody recently approved in our country for the treatment of osteoporosis. This review addresses the endocrine nature of bone, the role of osteocalcin, and specially, the regulatory and modulatory role of sclerostin on bone turnover and energy homeostasis through its inhibitory effect on canonical Wnt/β-catenin signaling, as well as its potential utility as a biomarker.

## Introduction

Bone is a specialized connective tissue with important functions. It serves as the structural support that enables the mechanical actions of soft tissues, such as muscle contraction or lung expansion, provides protection to vital organs (brain, heart and lungs) and bone marrow, and is the main reservoir of calcium and phosphorus in the body.

Ninety-nine percent of bone consist of an extracellular matrix primarily composed of hydroxyapatite carbonate. Type I collagen is the most abundant protein in the organic fraction of this matrix and other minority proteins include proteoglycans, matrix proteins: glycoproteins (bone sialoprotein, osteopontin, Dmp1, MEPE, etc.) and GLA proteins (osteocalcin, matrix GLA protein), as well as cytokines (IL1, IL6) and growth factors (TGFβ, BMPs). Different cells, coexist within this matrix including adipocytes, vascular smooth muscle cells, macrophages and mainly osteocytes, osteoblasts and osteoclasts. The latter three are directly involved in the metabolic activity of bone known as bone turnover.

Bone turnover is a dynamic process characterized by coordinated, sequential phases of osteoclastic resorption and osteoblast formation, induced and modulated by mechanical, hormonal and local factors. Osteocyte plays an essential role in the control of this activity by communicating with osteoblasts and osteoclasts through the expression of signaling proteins such as FGF23, the receptor activator of nuclear factor B ligand (RANKL), Dickkopf-1 (DKK1) and sclerostin. Turnover activity enables a continuous bone adaptation to the mechanical and biochemical needs of the body. It is estimated that the adult skeleton is completely renewed every 10 years [1]. The maintenance of this structure and its functionality demands a significant supply of energy and recent evidence suggests the involvement of some bone proteins, such as osteocalcin [2] or sclerostin [3] in the regulation of energy homeostasis to meet these energy requirements. The understanding of bone interaction with energy metabolism, along with the prior recognition of osteocyte FGF23 expression in phosphorus balance control, represents a revolution in the perception of bone, now considered the largest endocrine organ of the body.

This review discusses the endocrine nature of bone; the “bone-kidney-intestine-parathyroid” axis mediated by FGF23, and “bone-pancreas” connection mediated by osteocalcin. Lastly, it focuses on sclerostin, an osteocytic protein that regulates metabolic activity of bone tissue and modulates it with energy homeostasis through its interaction with the canonical Wnt/β-catenin signaling, a key element in the development and homeostasis of different organs in adulthood. As Wnt/β-catenin inhibitor, sclerostin is directly involved in the pathophysiology of different bone diseases, making it a potential biomarker and therapeutic target.

## Signaling pathways regulating bone metabolic activity

**Figure 1:**
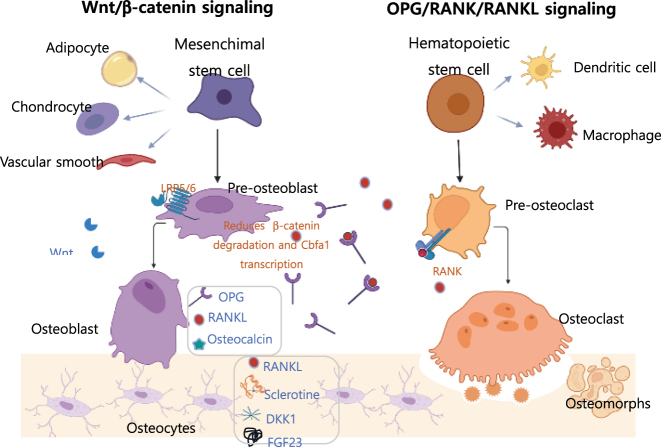
Bone remodeling signaling pathways. Wnt/β-catenin signaling is crucial in differentiation of mesenchimal stem cell into the osteoblastic lineage, which is a common precursor of chondrocytes, adipocytes and vascular smooth muscle cells. LRP5/6 activation by Wnt ligands reduces phosphorylation and degradation of β-catenin, thereby triggering the transcription of pro-osteogenic genes and osteoblastogenesis. Osteocytes, the final product of osteoblast differentiation, form a communication network that regulates bone remodeling via protein secretion (sclerostin, DKK1, RANKL y FGF23). Sclerostin and DKK1 inhibit Wnt signaling via interaction with LRP5/6. Although the main function of osteoblasts is to synthetise the extracellular matrix, they also secrete regulatory factors of osteogenesis such as RANKL and OPG. RANK signaling by RANKL promotes osteoclastic differentiation of the hematopoietic stem cell precursor (shared by macrophages and dendritic cells), fusion and activation of osteoclasts while it prevents their fusion into osteomorphs. OPG antagonizes this signaling by operating as a “decoy” receptor of RANKL and preventing its binding to the RANK receptor. Osteoclasts also release factors that stimulate bone formation (BMP6, Wnt10b). *Designed using BioRender.com.* LRP5/6, low density lipoprotein-receptor related protein 5 or 6; DKK1, Dickkopf-1; RANKL, receptor activator of nuclear factor kappa-Β ligand; FGF23, fibroblast growth factor 23; OPG, osteoprotegerin.

The molecular basis of bone turnover relies on interaction between two signaling pathways: the canonical Wnt/β-catenin signaling pathway, which regulates osteoclastogenesis, and the RANK/RANKL/OPG/(LGR4) pathway, which controls osteoclastogenesis (Figure 1).

### The canonical Wnt/beta-catenin signaling pathway

**Figure 2:**
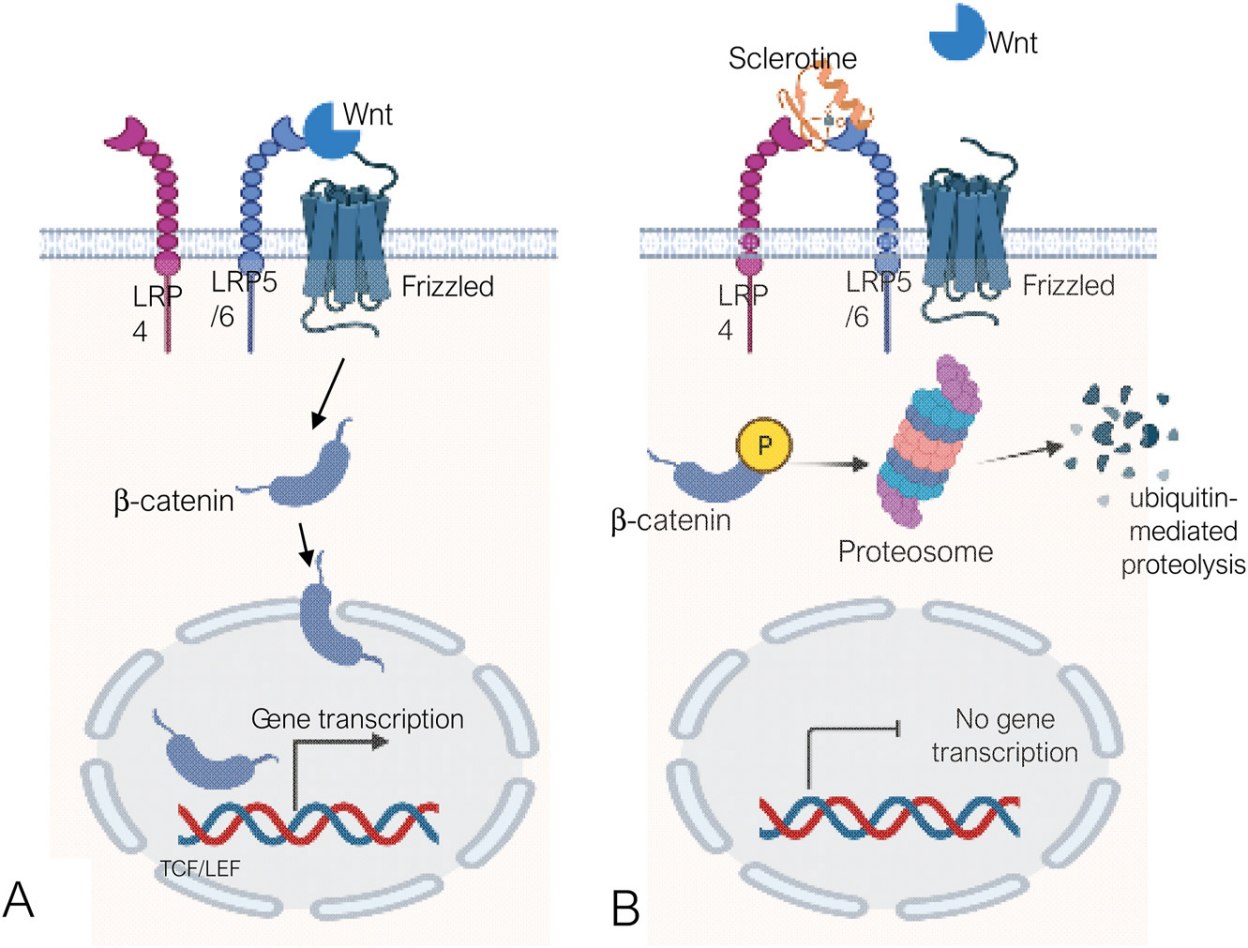
Wnt/catenin signalling and inhibition by sclerostin. Activation of the LRP5/6 receptor by Wnt ligands reduces β-catenin phosphorylation. This actions enables β-catenin translocation into the nucleus, where it displaces repressors, activates the transcription TCF/LEF complex and initiates pro-osteogenic gene transcription (including Cbfa-1β) [4], which stimulate osteoblast differentiation. (B) Inhibition of this signalling pathway is elicited by sclerostin via the LRP4 receptor, which “sequesters” this osteocytic protein to facilitate interaction with LRP5/6 and prevent its activation by Wnt ligands. When inhibited, β-catenin phosphorylates and degrades in the proteosome via ubiquitination, thereby preventing gene transcription. *Designed using Bio-render.com.*

The key element of canonical Wnt/β-catenin signaling is the regulation of the stability of β-catenin, which acts as a transcription factor. Its activation depends on Wnt ligands, a secreted cystein-rich glycoproteins, highly conserved throughout evolution and capable of activating the LRP5/6 receptor (low-density lipoprotein-receptor-related protein-5 or -6). In bone tissue, canonical Wnt/β-catenin signaling induces mesenchymal stem cell differentiation (common precursor of chondrocytes, vascular smooth muscle cells and adipocytes) into the osteoblastic lineage. Activation of the LRP5/6 receptor by Wnt ligands reduces β-catenin phosphorylation, enabling β-catenin translocation into the nucleus, where it displaces repressors, activates the TCF/LEF transcription complex and initiates pro-osteogenic gene transcription (including *Cbfa-1*) [4], which stimulate osteoblastic differentiation and bone formation (Figure 2A). In the absence of Wnt ligands, cytosolic β-catenin interacts with other components of the destruction complex (axin 1, APC, GSK-3, CK1), to be phosphorylated by the two kinases of this complex (GSK-3 and CK1) and degraded in the proteosome [5].

The relevance of this signaling pathway in bone development and adult bone homeostasis implies the need of a fine-tuned regulation by inhibitors and activators [6]. Among inhibitors there are proteins that block signaling by their interaction with LRP5/6 (DKK1 and sclerostin) (Figure 2B), proteins that interact with Wnt ligands to prevent their binding to LRP5/6 (secreted frizzled-related proteins [sFRPs] 1–5, Wnt1 inhibitor and secreted klotho) or even Wnt-induced factors that degrade LRP5/6 (ZNRF3). Conversely, Norrin and R-spondins (Rspo) are activators that enhance the interaction of Wnt ligands with LRP5/6 by binding to Wnt ligands. Pathologic conditions associated with impaired Wnt signaling lead to early-onset osteoporosis and a higher fracture risk, such as loss-of-function mutations in LRP5 (osteoporosis syndrome and pseudoglioma) or Wnt mutations [7, 8].

### The RANK/RANKL/OPG/(LGR4) system

Although the main function of osteoblasts is to synthesize the organic components of the bone matrix, they also regulate osteoclastogenesis through the secretion of a range of paracrine factors. These factors include RANKL, macrophage colony-stimulating factor (M-CSF), osteoprotegerin (OPG) and Wnt genes of the 5A (WNT5A) and 16 (WNT16) familiy. Among these, RANKL and OPG, both proteins belonging to the TNF family, play a crucial role in the differentiation and activation of osteoclasts. Osteoclasts, macrophages and dendritic cells are produced by the same pluripotential hematopoietic precursors. Their differentiation into the osteoclastic lineage requires the activation of the receptor activator of nuclear factor κB (RANK), located on the surface of osteoclasts and their precursors, by RANKL. RANK/RANKL interaction stimulates osteoclastic differentiation, fusion, adherence to bone and the activation of preexisting osteoclasts, while simultaneously inhibiting their apoptosis [9]. The expression of RANKL is under the control of hormones (parathyrine [PTH], calcitriol, glucocorticoids), cytokines (IL1, IL6, IL11) and growth factors (FGF and PDGF) [10]. The net increase in the number and activity of osteoclasts results in increased bone resorption through the secretion of acid and lithic enzymes (tartrate-resistant acid phosphatase and cathepsin K), which degrade the extracellular compartment. During resorption process, osteoclasts also release factors that stimulate bone formation (BMP6, WNT10B) or are involved in immune response (clastokines) [11].

Counteracting this signaling pathway, OPG synthetized in osteoblasts and stromal stem cells, acts as a decoy receptor by binding to RANKL and preventing its interaction with RANK [7]. This way, OPG inhibits osteoclast differentiation and activation while simultaneously promotes osteoclast apoptosis. However, recent studies suggest that, instead of this apoptotic process, osteoclasts may undergo fission process into smaller motile cells called osteomorphs capable of migrating and merging with another osteoclast [12]. The rapid fusion of these osteomorphs to form osteoclasts would partially explain the increased risk for fracture observed after discontinuation of Denosumab (an anti-RANKL, that mimics OPG). OPG expression is regulated by a variety of factors. Thus, it is increased by cytokines (TNF alpha, IL 1, IL18 TGF beta), morphogenetic proteins (BMPs) and steroid hormones (17beta estradiol) and decreased by glucocorticoids, PTH and E2 prostaglandin [11].

RANK has traditionally been considered the only RANKL receptor. However, at least one additional receptor was recently identified: LGR4 (leucin-rich-repeat-containing G-protein-coupled receptor 4), which competes with RANK suppressing it signaling during osteoclast differentiation. By binding to RANKL, the soluble extracellular domain of LGR4 downregulates osteoclast differentiation and bone resorption *in vivo* [13]. Recent evidence suggests that LGR4 is involved in the development of multiple organs and plays a role in immune processes, metabolic diseases, and carcinogenesis [14].

### The role of osteocyte in the control of these signaling pathways

Osteocytes, the most differentiated cells of the osteoblast lineage account for 90–95 % of total bone cells. These cells are arranged in a reticular structure within the mineralized bone matrix to form a communication network that responds to mechanical and hormonal stimuli by regulating and coordinating osteoblast and osteoclast function. Therefore, osteocytes play a major role in the control of bone remodeling and mineralization acting as endocrine cells that synthesize key molecules such as RANKL, DKK1 and sclerostin. These signaling proteins would potentially be used at future in the clinical management of metabolic bone disease, as they reflect specific biological processes within bone and its interaction with other tissues.

## The bone: a large endocrine organ

All cells present in bone tissue, including mesenchymal stem cells and adipocytes, can synthesize and secrete a wide variety of bioactive molecules, such as proteins, polypeptides, cytokines, inflammatory factors, adipokines (leptin, adiponectin) and even exosomes, to regulate bone remodeling. In parallel, the lacuno-canicular arrangement of bone provides an important route of exchange with circulation; the presence of these bone factors in the bloodstream suggests their potential extraosseous action. Even more, beyond the paracrine activity of bone cells, evidence supports the action of these factors released into circulation on distant organs, further confirming the endocrine nature of skeleton.

### FGF23: bone-intestine-kidney-parathyroid gland connection

The identification of FGF23 in familial hypophosphatemic rickets [15] has been a step forward in our understanding of mineral homeostasis. Bone tissue is no longer considered a simple target organ, and it emerges as an endocrine organ that directly connects the bone with the intestine, kidneys and parathyroid gland through this osteocytic protein. FGF23 is, along with its co-receptor klotho, the main regulatory hormone of phosphorus balance [16]. FGF23 protects the body from harmful chronic phosphate retention by its phosphaturic action and its suppressive effects on calcitriol and PTH. At present, FGF23 represents an interesting biomarker in conditions such as chronic kidney disease (CKD), where secondary hyperphosphatonism, despite being an adaptive mechanism, has negative effects on the body [17]. Moreover, it is a valuable diagnostic tool in primary disorders associated with FGF23 overproduction, such as hypophosphatemic rickets or tumor-induced osteomalacia, where it also serves as a therapeutic target following the development of an anti-FGF23 antibody (Burosumab) [18]. Although its main function is phosphate control, other additional actions have been suggested for FGF23, including effects on iron metabolism, inflammation, erythropoiesis and even cognitive functions [19]. At paracrine level, a connection between FGF23 and some bone remodeling abnormalities, such as Wnt1 mutations leading to early-onset osteoporosis and increasing fracture risk, has been suggested. Despite severe bone involvement, classical bone turnover markers (BTMs) and Wnt signaling inhibitors (DKK1 and sclerostin) remain unaltered, being circulating FGF23 the only elevated biomarker [20].

### Osteocalcin: the bone-pancreas-energy metabolism connection

Shortly after the recognition of bone as an endocrine organ involved in mineral homeostasis, Karsenty et al. observed by genetic manipulation in mice that, just as bone tissue is affected by hormones such as adiponectin, leptin and insulin, there is a feedback mechanism in the control of energy metabolism by osteocalcin [2]. Osteocalcin, a protein primarily expressed by osteoblasts (it serves as marker of osteoblastic differentiation in cell cultures) is the most abundant non-collagenous protein in bone matrix. It is a gamma-carboxyglutamic acid (GLA) protein with three gammacarboxyglutamic residues (at C17, 21 and 24), being this carboxylation vitamin K-dependent. This conformational change increases its affinity for calcium and, by binding hydroxyapatite crystals, it establishes bridges between the mineralized matrix and collagen. Osteocalcin promotes bone formation and remodeling (it is a bone formation BTM), stabilizes bone tissue, improves its mechanical properties and prevents fractures. Part of this osteocalcin is either not fully carboxylated or is decarboxylated in the acidic environment during bone resorption, lossing its affinity for bone tissue, and enters into circulation to act as an endocrine factor. This undercarboxylated or non-carboxylated osteocalcin is the active circulating form, accounting for 40–60 % of total serum osteocalcin and its measurement is considered an indicator of Vitamin K status.

**Figure 3:**
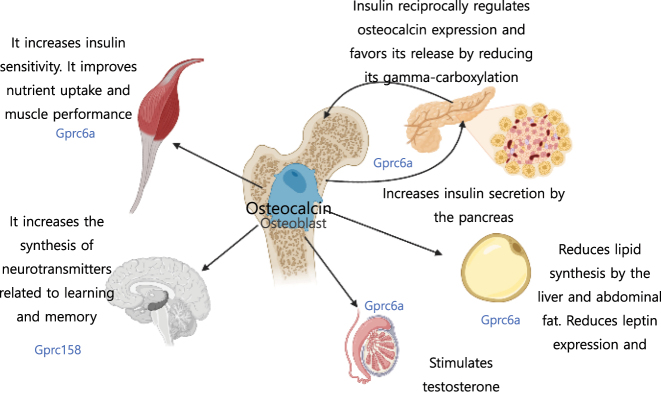
Endocrine activity of osteocalcin. Under-carboxylated osteocalcin loss affinity for the bone and is released into the circulation to exert endocrine functions, possibly via Gprc6a and Gprc158 receptors. It is involved in energy homeostasis and induces pancreatic β cells and insulin secretion, reducing triglycerides synthesis and body fat by the liver, reducing leptin expression and increasing adiponectin expression in adipocytes. Reciprocally, insulin regulates osteocalcin expression in osteoblasts and favors its release by reducing carboxylation. Osteocalcin also acts on skeletal muscles by improving their performance. In animal models, osteocalcin has been demonstrated to exert effects on Leydig cells by increasing the synthesis of testosterone. *Designed using BioRender.com*.

This research group demonstrated that mice lacking osteocalcin display reduced beta cell proliferation in the pancreas, glucose intolerance, and insulin resistance [2]; a phenotype that is corrected by the administration of osteocalcin. This hydrocarbonate intolerance phenotype is associated with increased visceral fat, elevated triglycerides levels, reduced adiponectine and higher leptin levels. Subsequent studies have demonstrated that insulin receptor signaling in osteoblasts is needed for the synthesis of osteocalcin, suggesting the existence of a bone-pancreas feedback loop by which insulin signaling would regulate osteocalcin expression and promote its release by enhancing its undercarboxylation [21]. Alongside its effects on pancreatic beta-cell proliferation and insulin secretion, osteocalcin has been found to exert other effects related to energy metabolism, including effects on the intestinal epithelium (incretin synthesis), hepatocytes and adipocytes [22], [23], [24]. Osteocalcin also acts on skeletal muscles (enhancing nutrient uptake and performance); brain (increasing the synthesis of neurotransmitters related to learning and memory); parasympathetic neurons (regulating the acute stress response); and testes (stimulating testosterone production) (Figure 3). These actions are probably exerted via Gprc6a, a receptor expressed by adipose tissue, skeletal muscles and Leydig cells that allows osteocalcin to operate as a nutrient sensor, and via Gpr158 in the central nervous system [25, 26]. However, reduced circulating levels of osteocalcin in men treated with zoledronic acid are not associated with changes in levels of testosterone, suggesting that osteocalcin may not affect Leydig cell function in adult men [27]. It is worth mentioning that the characteristic metabolic phenotype of Karsenty’s murine model has not been observed in other osteocalcin-null models. These discrepancies may be due to methodological limitations (method of ablation, feeding, microbiome…) along with the complexity of ostocalcin in mice, as it is encoded by three genes (Bglap, Bglap2 y Bglap3). However, Karsenty’s model has been validated by nearly 2,000 studies carried out in mouse models of gain-of-function mutations, injections of recombinant protein and *in vitro* assays [26]. Unlike mice, osteocalcin is encoded in humans by a single gene (*BGLAP*) and the measurement of its active undercarboxylated form remains a technical challenge due to the low sensitivity of current methods [28]. Nevertheless, there is cumulative evidence of the association between osteocalcin and energy metabolism, especially in type 2 diabetes mellitus [29].

In addition to osteocalcin, lipocalin 2 (LCN2), a ubiquitously expressed protein associated with increased osteoclastic activity and bone resorption, is also associated with elevated insulin secretion and sensitivity, glucose tolerance and even control of appetite. Initially believed to be exclusive to adipose tissue, LCN2 is expressed 10 times more in osteoblasts than in adipose tissue or other organs [30].

## Sclerostine: a new osteocytic biomarker

Recently, moreover after the identification of FGF23, the role of osteocyte is being revaluated as its large reticular structure allows it to “sense” and “respond” by releasing different factors that operate at paracrine and endocrine level; among them is sclerostin.

### Sclerotine: a natural brake of the bone

Sclerostin is a 22kd glycoprotein of 213 amino acids encoded by the *SOST gene.* It is primarily secreted by osteocyte and serves as a natural brake by preventing bone overgrowth. Sclerostin was first described in two rare genetic diseases characterized by loss-of-function mutations in *SOST* gene: sclerosteosis [31] and Van Buchem disease [32]. Both disorders exhibit a sclerostin deficiency associated with a significant increase in bone mass, hyperostosis, and foraminal stenosis that leads to cranial nerve entrapment resulting in deafness and facial palsy.

The osteocyte reticular network within the mineralized matrix represents a mechanosensor that allows the bone to “sense” and “react” to mechanical strain. The perception of mechanical stimuli inhibits *SOST* expression and sclerostin secretion by osteocytes, maybe trough a signaling pathway in which prostaglandin E2 (PGE2) is involved, thereby triggering an osteo-anabolic response [33]. By contrary, maintained absence of mechanical stimuli, as it occurs under lack of gravity situations during spaceflights, induces a continuous *SOST* activation and sclerostin overexpression that would lead to bone mass loss higher than 20 % after a space mission [34].

Along with this mechano-sensitive response, the osteocytic network is also sensitive to hormonal stimuli, to coordinate the bone turnover activity with the mineral homeostasis maintenance. Calcitriol induces sclerostin expression in osteocytes, but this association is controversial because the increase in sclerostin levels induced by calcitriol has been solely observed in men [35]. Of note, the effects of 1.25(OH)_2_D_3_ on sclerostin expression in humans differ from those in mice and moreover, it has been suggested differences in the mechanism of action between endogenous calcitriol (inhibition of sclerostin expression) and the exogenous form or its analogs (promotion of sclerostin expression) [33]. Conversely, estrogens reduce sclerostin expression, as demonstrated by several studies in women treated with transdermal estradiol patches [36], which partially explains the protective effect of estradiol on bone mass. This suppressive effect of estradiol on *SOST* is mediated by beta receptor, while the alpha estrogen receptor promotes the opposite effect [37]. Finally, PTH is an important inhibitor of *SOST* and sclerostin expression [38]; in fact, the bone anabolic effect of intermittent administration of PTH is supported by this suppressive action. The infusion of 1–34 PTH induces a significant reduction of sclerostin at 6 h that persists for 18 h [36]. This rapid decrease of sclerostin following the administration of PTH suggests that, along with the suppression of *SOST* transcription, PTH would also induce the lisosomal degradation of sclerostin [33].

### Paracrine actions of sclerostin

**Figure 4:**
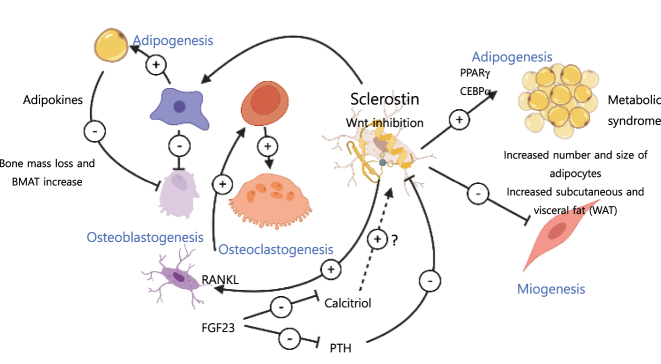
Paracrine and endocrine activity of sclerostin. At paracrine level, the blocking of Wnt/β-catenin signaling by sclerostin inhibits differentiation into the osteoblastic lineage of the mesangial stem cell and induces differentiation into an adipogenic form, resulting in an increase of BMAT. Adipokine secretion by the adipose tissue further modulates the negative effects of sclerostin on osteoblastogenesis and bone formation. In the osteocyte, sclerostin increases both, RANKL expression by the osteocyte, which promotes osteoclastogenesis and bone resorption, and FGF23 transcription, which exerts a suppressive effect on Vitamin D and PTH (which inhibits sclerostin expression). At endocrine level, Wnt inhibition by sclerostin induces adipogenesis in master regulators PPAR and CEBPα, thereby suppressing fibroblastic differentiation and myogenesis. These events increase body fat and the number and size of adipocytes and augment the incidence of metabolic syndrome. The perception of a mechanical stimulus suppresses sclerostin expression, triggering the pro-anabolic and anti-catabolic response of Wnt signaling by reducing RANKL expression in bone osteocytes. To meet the biochemical (calcium and phosphorus) and energy requirements of this osteo-anabolic response, FGF23 synthesis in the bone decreases, thereby reducing its phosphaturic and suppressive effect on PTH and vitamin D and reduces *de novo* fatty acid synthesis. *Designed using BioRender.com.* BMAT, bone marrow adipose tissue; WAT, white adipose tissue.

Sclerostin exerts a direct anti-anabolic action on bone tissue by binding low-density lipoprotein-receptor-related protein (LRP4); in fact, some cases of sclerosteosis are caused by mutations in this protein [39]. LRP4 “sequesters” sclerostin and facilitates its interaction with Wnt LRP5/6 co-receptors, thereby avoiding their binding to Wnt1 and Wnt3a. The binding of sclerostin to LRP5/6 promotes β-catenin phosphorylation and degradation in the proteosome, leading to suppression of pro-osteogenic gene transcription and osteoblastogenesis (Figure 2B). Along with this direct inhibitory action on canonical Wnt/β-catenin signaling, sclerostin indirectly promotes osteoclastogenesis and bone resorption by inducing RANKL expression and secretion by osteocytes [40] (Figure 4).

Suppression of Wnt-signalling in bone tissue drives the differentiation of mesenchymal stem cell towards non-osteogenic lineages inducing, among others, adipogenesis. Bone marrow adipose tissue (BMAT) has attracted widespread interest from the scientific community. Its anatomical location makes it possible a potential “fat-bone” axis, in which adipocyte would modulate bone turnover through the secretion of adipokynes and cytokines. BMAT accounts for 50–70 % of the bone marrow in healthy adults, it increases with age and is inversely associated with bone mass. Thus, BMAT increases in patients with low bone mass conditions, such as osteoporosis or anorexia nervosa [41]. It has been shown that sclerostin promotes the adipocyte differentiation of pre-adipocytes cell lines, which explains the reduction of BMAT exhibited by sclerostin-null rats [42]. Likewise, the administration of anti-sclerostin antibodies reduces BMAT depots, with a decrease in the number and size of adipocytes. In humans, a direct relationship has been observed between elevated levels of sclerostin and BMAT in CKD patients, suggesting a possible regulatory role of sclerostin on BMAT [41].

### Endocrine role of sclerostin on mineral metabolism (Figure 4)

The *Sost* ^*−/−*^ mouse represents an ideal model for examining interaction between sclerostin suppression and mineral balance and determine the potential endocrine activity of sclerostin. The high bone apposition rate of these mice requires a positive calcium/phosphorus balance. Thus, this transgenic model shows a net increase in calcitriol values secondary to a higher expression of renal 1alpha-hydroxylase and a reduction in 24-hydroxylase expression. In these models, an increase of tubular calcium reabsorption is also observed, although circulating calcium levels are not elevated (probably due to bone sequestration), and PTH remains within the reference interval [43, 44]. Along with this calciotropic activity, a decrease in circulating levels of FGF23 is also observed, preventing a potential negative balance of phosphate caused by its phosphaturic and inhibitory effects on vitamin D.

The problem is that studies on mineral homeostasis in animal models of sclerostin overexpression are scarce, limiting our understanding about the endocrine role of sclerostin on it. However, a study in an osteocyte cell line (IDG-SW3) (expressing FGF23 at mRNA and protein level) demonstrates that the administration of sclerostin induces the synthesis of FGF23 by suppressing the expression of negative regulators of its transcription (PHEX and DMP1) and simultaneously reduces its proteolytic degradation through the up-regulation of GALNT3 expression and stabilization of the FGF23 molecule [45].

### Endocrine actions of sclerostin on energy metabolism (Figure 4)

Wnt signaling suppresses the expression of master-regulators of adipogenesis (PPARγ and CEBPα), thereby promoting fibroblastic differentiation and myogenesis. The inhibitory effect of sclerostin on Wnt/β-catenin also affects subcutaneous and visceral white adipose tissue (WAT) and energy metabolism. *Sost* ^−/−^ mice have lower overall fat mass and reduced mass of white adipose tissue from gonadal, inguinal and retroperitoneal pads, with a decrease in adipocyte number and size. At the same time, these mice generate resistance to fat-rich diet and show a decrease in circulating levels of free fatty acids and glucose, related to higher fatty acid oxidation and reduced *de novo* fatty acid synthesis. Conversely, sclerostin overexpression induces an increase in whole body fat mass, with an increase in number and size of adipocytes in white adipose tissue [3]. This pro-anabolic and anti-catabolic activity of sclerostin on adipocytes is a direct effect, since the ablation of LRP4 in adipose tissue reduces adipocyte hypertrophy *in vitro* and *in vivo*, improves lipid metabolism, and increases insulin sensitivity. Moreover, the selective inhibition of LRP4 in osteoblast causes an overexpression of circulating sclerostin overexpression and the opposite metabolic effect [46].

The adipogenic activity of sclerostin resulting from Wnt inhibition has also been demonstrated in humans. Individuals with a gain-of-function mutation in LRP5 and high bone mass phenotype show lower body fat depot. Inversely, individuals with loss-of-function mutations in LRP5 and low bone mineral density mass exhibit increased abdominal fat mass [47]. Of note, LRP5 expression is higher in subcutaneous than visceral WAT and this would modify the body fat distribution. Moreover, biopsies from healthy volunteers register that LRP5 expression is higher in abdominal subcutaneous WAT than in gluteo-femoral fat, affecting the metabolic risk (adipokines secreted by abdominal fat have a less favorable profile). Clinical studies demonstrate a positive correlation between sclerostin, fat mass and the incidence of metabolic syndrome. Patients with type 2 diabetes exhibit significantly higher levels of serum sclerostin, than non-diabetic controls [48, 49]. However, these results should be interpreted with caution, due to errors in the accuracy and consistency of methods used to measure sclerostin and, in fact, their results differed significantly depending on the measurement technique used [50]. Even more, it is unclear to what extent the circulating sclerostin represents the active protein.

### Sclerostin as a biomarker and therapeutic target

Circulating sclerostin levels are higher in men than in women at any age, possibly as reflect of the largest bone mass of the former, since the main source of sclerostin is the osteocyte pool. This would explain the positive correlation between circulating sclerostin and bone mineral density registered in most studies, although high levels of circulating sclerostin would be expected to be associated with a low bone density [51]. Sclerostin is higher in postmenopausal than in premenopausal women and it increases with age, maybe due to the contribution of extraosseous tissues such as kidney or vascular calcification, since the levels of sclerostin mRNA in the bone remain constant at all ages [52].

In CKD, circulating levels of sclerostin increase as glomerular filtrate rate decreases, reaching up to four-fold its predialysis value [53, 54]. This increase is not explained by the loss of clearance, since its small molecular size allows filtration and reabsorption. This is supported by a study that demonstrated that renal excretion of sclerostin increases as kidney function declines [55]. A range of studies suggest an initial increase of sclerostin synthesis by the osteocyte during the course of CKD, but the subsequent increase of PTH inhibits *SOST* and sclerostin expression [54, 56] and in fact, sclerostin correlates negatively with PTH and bone turnover markers. The concurrent increase of circulating levels of sclerostin increase with the decrease of glomerular filtrate rate could also have an extraosseous origin such as vascular calcification, a prevalent condition of bone and mineral disorders associated to CKD (MBD-CKD). Vascular calcification is a complex process in which vascular smooth muscle cells undergo a phenotypic transformation into osteoblast-like cells with ability to express sclerostin. Therefore, inhibition of the Wnt/β-catenin signaling pathway at vascular level could represent a defense mechanism by which sclerostin would play a protective role against vascular calcification [57, 58].

Sclerostin is also found increased in other bone diseases, such as Paget’s disease or lithic bone metastases. In multiple myeloma, the increase in the apoptosis of osteocyte leads to RANKL and sclerostin overexpression. Increased levels of sclerostin in these patients are associated with poorer survival rates [59]. Finally, an association between sclerostin and neurodegenerative diseases, including Alzheimer’s disease, has been suggested based on a possible pleiotropic activity of Wnt/β-catenin signalling on synaptic plasticity and memory. However, further studies are needed to confirm this hypothesis [60].

Therefore, sclerostin emerges as a potential biomarker to be used in the clinic to asses bone disorders and their interaction with other tissues, as supported by recent studies [61, 62]. However, the lack of standardization of the techniques used to measure sclerostin limit its clinical usefulness [51]. Methods currently available show important discrepancies on accuracy and specificity which influences their interpretation and explain the inconsistent results across studies. Only three methods measure the intact form of sclerostin: two ELISA techniques (Biomédica and TECO medical) and the only automated method (Diasorin), and none of them has been validated for clinical use. The harmonization of these methods in the future is crucial.

In parallel to its importance as potential biomarker, an anti-sclerostin antibody has currently been developed as a therapeutic target: Romosozumab (AMG785), a humanized monoclonal IgG2 that binds to and neutralizes the action of human sclerostin. The use of this antibody in Europe was approved in 2020 and recently in Spain. This antibody exerts a dual mechanism on bone cells: pro-anabolic and anti-resorptive, showing a rapid restoration of bone mineral density and a significant reduction in fracture risk. A recent safety and systematic review on the use of sclerostin for the management of osteoporosis reveals increases in bone mineral density between 13.1 and 13.3 % at lumbar level, 2.2–5.9 % and 2.5–6.9 % at femoral neck and total hip, respectively, along a reduction of vertebral, main and hip fractures [63]. Romosozumab is not only a promising agent for the management of osteoporosis, but is also exerts positive effects on bone involvement in multiple myeloma, where increased levels of sclerostin may contribute to the development of lytic lesion.

## Conclusions

The change of paradigm in mineral homeostasis also involves the bone. The role of osteocyte as endocrine cell is gaining relevance by its involvement in the control of bone turnover and phosphate balance through the synthesis of key molecules (DKK1, sclerostin, FGF23), as well by its relationship with BMAT, a tissue that is arising great interest. Beyond their paracrine activity, various osteocytic and osteoblastic proteins are also involved in energy homeostasis by controlling the supply and storage of nutrients, insulin secretion, and fat depots, and regulating energy consumption in parallel to turnover activity. Through these “osteokynes”, the bone interacts with other organs including pancreas, liver, or skeletal muscle by modulating energy consumption and connecting physical activity with bone health, and even with the brain by their involvement in appetite control and cognitive functions.

Although the role of these osteokynes in humans has been confirmed on different clinical trials, further studies are needed to validate them, since much of our knowledge is derived from animal models. There are technical limitations due to the low sensitivity of assays available to measure undercarboxylated osteocalcin in humans, along with discrepancies on sclerostin results by the lack of standardization and low specificity of methods. The development and harmonization of reliable methods will contribute to define the role and usefulness of these proteins as bone biomarkers for the clinical management of different pathologic conditions, where the clinical laboratory will play a key role.

Along with its importance as a biomarkers, the knowledge of these connections between bone and other tissues raises therapeutic hypothesis, such as possible beneficial effects with the exogenous administration of osteocalcin on insulin secretion in sarcopenia. In fact, FGF23 and sclerostin are currently being used as therapeutic targets after the development of antibodies against these proteins for the treatment of XLH and osteoporosis, respectively. This is just the beginning of the hidden cross talk between the bone and other tissues.
